# Vascular spinal cord obstruction associated with superior vena cava syndrome: A case report and literature review

**DOI:** 10.1097/MD.0000000000009196

**Published:** 2017-12-22

**Authors:** Xiaoling Zhang, Xinyuan Li, Meihong Meng, Jie Cao, Xiaonan Song, Kangding Liu, Shaokuan Fang

**Affiliations:** Department of Neurology, Neuroscience Centre, the First Teaching Hospital of Jilin University, Changchun, China.

**Keywords:** esophageal cancer, spinal vascular diseases, superior vena cava syndrome, vascular spinal cord obstruction, vertebral venous system

## Abstract

**Rationale:**

Superior vena cava syndrome (SVCS) is the obstruction of blood flow through the SVC, causing complete or partial blockade of the collateral circulation of returning venous blood. SVCS is frequently presented with facial, neck, trunk, and upper limbs swelling and so on. However, to the best of our knowledge, the obstruction of the venous return in the spinal veins is rarely a manifestation of SVCS.

**Patient concerns:**

We presented a rare case of a 52-year-old male patient with 2-month history of progressive right upper limb numbness and swelling and 10-day history of extremities malfunctioning. Cervical magnetic resonance imaging (MRI) detected obstruction of the spinal venous return. Lung computed tomography (CT) revealed lesions in the esophagus, which indicated esophageal cancer with mediastinal lymph nodes metastasis and signified SVCS.

**Diagnoses:**

With the results of laboratory findings, cervical MRI, lung CT findings, and physical examination, the patient was diagnosed with SVCS manifesting as spinal vein obstruction.

**Interventions and outcomes:**

The family abandoned further treatment, and the patient passed away 2 months after discharge.

**Lessons:**

The case indicates that SVCS can induce systemic and spinal cord diseases affecting the venous return. Further studies are necessary to reveal the mechanism for SVCS inducing spinal veins obstruction and to explore whether SVCS patients with and without vascular spinal cord obstruction have different prognoses.

## 1 Introduction

Superior vena cava syndrome (SVCS) is the obstruction of blood flow due to most frequently intrathoracic malignancy and rarely intravascular devices,^[1]^ causing complete or partial blockade of the collateral circulation of returning venous blood. The common clinical symptoms include facial, neck, trunk, and upper limbs swelling, difficulty in breathing and swallowing, chest pain, coughing, lightheadedness, visual symptoms, glottis edema, pleural effusions, as well as hoarseness.^[2]^ However, SVCS is rarely observed to induce spinal vein obstruction. Herein, we report a case of a 52-year-old male patient with spinal vein blockage as a complication of SVCS.

## 2 Ethic statement

This study was approved by the ethics committee of the First Hospital of Jilin University, Changchun, China. Written informed consent was obtained from the patient.

## 3 Case presentation

We presented a case of 52-year-old male who complained of progressive right upper limb numbness and swelling over 2 months and extremities malfunctioning over the previous 10 days. Past medical history included a 10-year history of hypertension, along with smoking and drinking habits for more than 20 years. He reported that such right upper limb numbness occurred after carrying heavy objects. Subsequently, he successively suffered right arm and right chest pain and swelling, laborious performance of bilateral upper limbs (more severe in the right side), dysfunction of lower limbs, and then urinary and defecation disorders. The results of physical examination were as follows: conscious, obese, passive position, right arm and right chest swelling, normal vital signs. Neurological examination revealed right eye enophthalmos, right upper eyelid ptosis, miosis (constriction of the pupil), and right-sided anhidrosis (lack of sweating). Motor examination showed weak limbs muscle strength: right upper limb (manual muscle test, lower extremities (manual muscle test), and weak left grip strength. Sensory examination showed that the pin-prick sensation was decreased below bilateral T2 level and the lower extremities joint position sense and tuning fork vibration sense were lost. Positive Babinski sign was observed on the left side. Note that Babinski sign was suspected to be positive on the right side; however, the symptoms were unclear and, thus, conclusions cannot be drawn.

**Figure 1:**
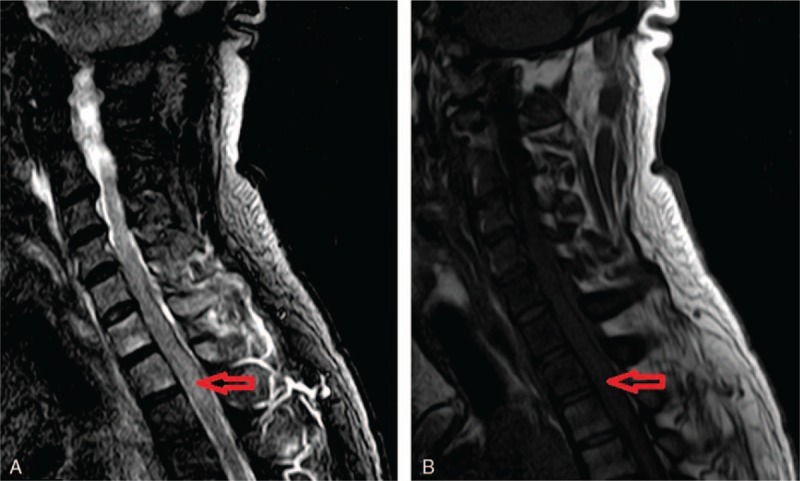
MRI of the cervical spinal cord. A, The T2-weighted images showing uneven increased signal intensity at C4 to T1 level (red arrow). B, The T1-weighted images showing obvious spinal cord thickening at C4 to T1 level (red arrow). MRI = magnetic resonance imaging.

**Figure 2:**
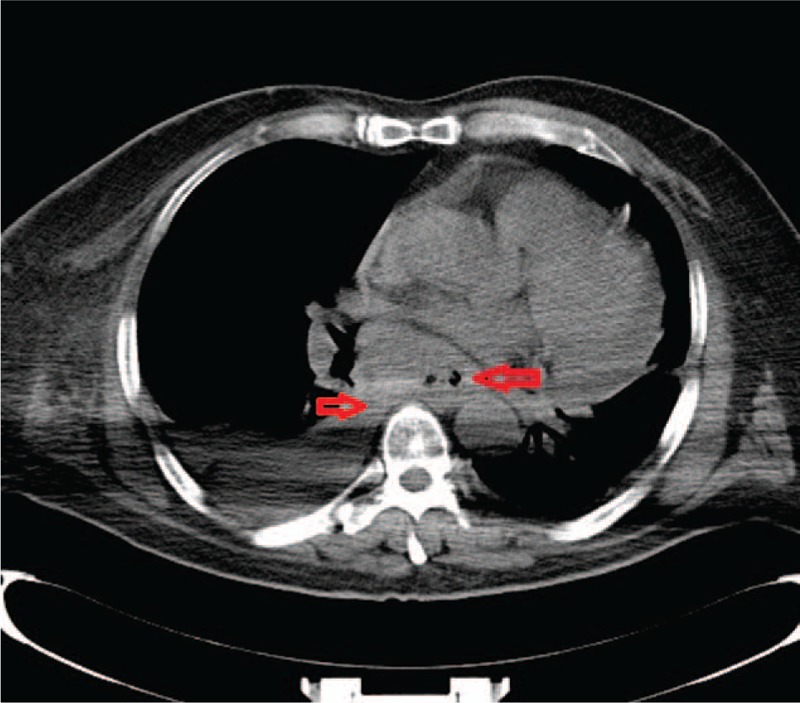
The mediastinal window of the lung CT. The mediastinal window of the lung CT showing multiple mediastinal lymph nodes (blue arrow) and alteration of the lower esophagus (red arrow). CT = computed tomography.

The results of laboratory routine examinations including complete blood count, urine, coagulation, electrolytes, liver function tests, fasting blood glucose, and serum lipid were normal. The concentrations of tumor markers were as follows: cytokeratin 19 fragments, 136.29 ng/mL (normal references, <5.00 ng/mL) and carcino-embryonic antigen 125, 129.89 U/mL (normal references, <10.00 U/mL). The neck vein ultrasound scanning on day 7 before admission detected venous thrombosis in the right internal jugular vein and right subclavian vein. Cervical magnetic resonance imaging (MRI) was performed 2 days previously, on day 9 before admission, and spinal cord thickening was observed at C4 to T1 with uneven increased signal intensity (Fig. 1). Head MRI performed on the same day (day 9 before admission) revealed left corona radiata lacunar cerebral infarction without new lesions. Lung CT was performed on day 1 after admission. The CT images revealed multiple mediastinal lymph nodes and alteration of the lower esophagus possibly due to esophageal cancer with mediastinal lymph node metastasis, which may result in SVCS (Fig. 2). Overall, the patient was diagnosed with SVCS, spinal vein obstruction, and esophageal lesions. Moreover, an esophageal biopsy was necessary to be performed for further detailed diagnosis. After discussion with the patient's family, the family abandoned further examination and treatment, and the patient was discharged. After discharge, the symptoms progressed, and the patient died of difficulty in breathing 2 months later.

## 4 Discussion

**Figure 3:**
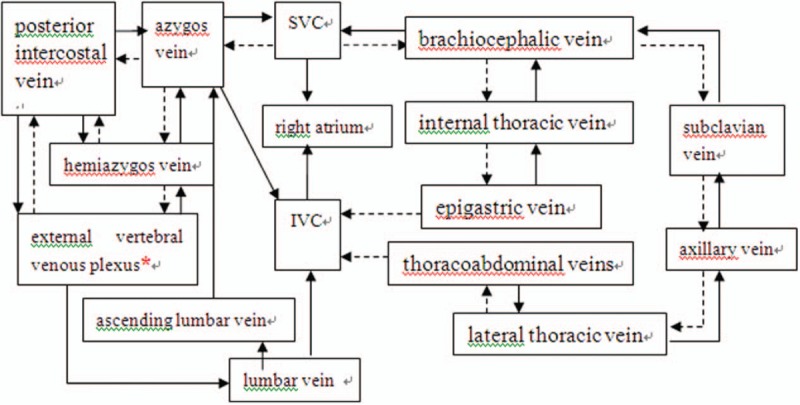
Primary collateral circulation for SVC obstruction above the azygos arch. Arrows indicate the physiological collateral pathway while dotted lines indicate converse collateral pathway to inferior vena cava for SVC obstruction above the level of azygos arch. SVC = superior vena cava.

Almost one-third of the venous blood flows back into the heart via the SVC, specifically, the returning blood from the head, arms, and upper torso.^[3]^ The SVC is vulnerable to compression and traction by the anterior mediastinum, the mediastinal mass, right bronchus, and parasternal lymph nodes due to its anatomical location and the intrinsic thin-wall and low blood pressure characteristics.^[3]^ Since the pressure increases when vena venous blood flow is blocked, obstruction of the vein may cause dilation of many subsidiary veins including the azygos vein, hemiazygos vein, intercostal vein, and so on. Furthermore, the azygos vein connects SVC with inferior vena cava and the azygos arch can be considered the center where SVC obstruction may be subdivided into 2 subtypes above and below the level of the arch. Therefore, the obstruction of different parts of the collateral circulation would result in different complications.^[3]^ Respectively, blood would run through the azygos vein into the SVC or some other collateral vessels to the IVC and then to right atrium when SVC obstruction happens above the level of azygos arch^[4]^ (as shown in Fig. 3). For the lower section obstruction, the blood flows mainly through azygos vein to hemiazygos vein, the inferior vena cava, and finally the right atrium.

After the arterial network surrounding the spinal cord enters the intramedullary space, a spinal cord capillary bed is formed. Subsequently, blood in the radiation-like intramedullary venous and sulcal veins within the pia mater would drain into the perimedullary vein and the perimedullary vein can be divided into anterior and posterior spinal veins connected via reticular veins, and thus, they were called the coronary veins.^[5]^ There are numerous anastomoses between the deep and superficial venous drainage systems and between the anterior and posterior median spinal veins, especially in the region of the thoracolumbar enlargement.^[6,7]^ Blood from the perimedullary veins would flow through the limited number of radicular veins to internal vertebral and finally to external vertebral venous plexus, traversing the intervertebral foramen above and below the pedicle between the upper and lower cervical levels,^[7]^ between the upper and middle chest, as well as between the lower chest and waist, respectively.^[8]^ The blood flows from the internal vertebral venous plexus to external vertebral venous plexus (as shown in Fig. 3) through intervertebral veins (Table 1).

**Table 1:**
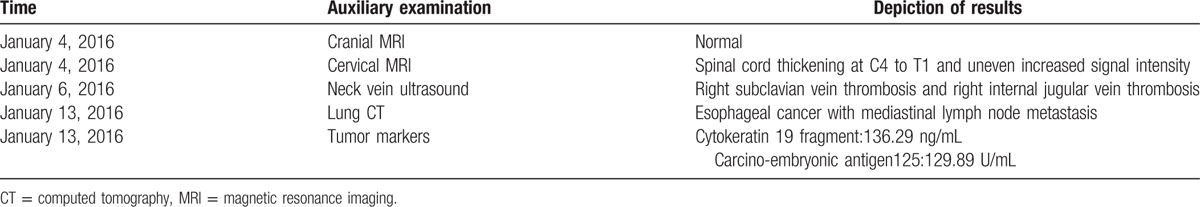
Depiction of important milestones related to the diagnoses.

The patient may have suffered from SVC compression in the SVC above the level of azygos arch, causing azygos vein obstruction. As a result, venous blood from the external vertebral venous plexus returned ineffectively. Moreover, the compression of the upper and lower cervical spinal cords might occur due to the engorgement of perimedullary veins secondary to enlarged external vertebral venous system. Because there were more abundant anastomoses around the thoracic and lumbar spinal cord than the cervical spinal cord,^[6,7]^ our patient only had spinal cord thickening at C4 to T1. Furthermore, the spinal venous disorders were gradually produced by the esophagus tumor. Hence, the only symptom was numbness of the right upper limb at the early stage. Gradually, the disease progressed to involve the upper and lower cervical spinal cord segments, which were evident from the cervical spine MRI (Fig. 2). Ultimately, the disease progressed and symptoms of motor, sensory, horner syndrome, and urinary and defecation disorders were observed. Our patient had positive pathological sign, which indicated that the pyramidal tract was damaged. Actually, the left corona radiata lacunar infarction may also induce paralysis on the right limbs and the right side positive Babinski sign, which made the diagnosis of the patient more complicated. However, the successively emerged symptoms, such as the right arm and right chest swelling, dysfunction of the left limbs, and urinary and defecation disorders, support our final clinical diagnosis. Postmortem examination of the spinal cord would have been informative. Unfortunately, the patient's family refused further examinations. The vertebral venous plexus route is 1 of 4 main collateral routes when the SVC is obstructed (as shown in Fig. 3) and these veins are devoid of valves, permitting bidirectional blood flow.^[9]^ Therefore, SVCS rarely manifests as obstruction of the spinal veins. It should be noted that our patient's right upper limb numbness occurred after carrying heavy objects. In this process, the vertebral venous plexus route was the only contributing route on account of its anatomical structure, while the increased abdominal pressure affected the other 3 collateral routes. In this way, the blood flowing through the vertebral venous plexus route markedly increased, so that the cervical cord veins congested, because of less abundant anastomoses. Our patient also showed one of the common SVCS manifestations as swelling in the right upper limb and chest. Unfortunately, the patient rejected spinal magnetic resonance venography (MRV) or digital subtraction angiography (DSA) to confirm the cause of myelopathy, and pathological biopsy of the esophagus contributing to better diagnosis. Since intravascular ultrasound (IVUS) might provide a more accurate representation of vessel compression and measurements than DSA,^[10]^ DSA for diagnosis of SVCS was not necessary. However, further pathological biopsy of the esophagus was required to be conducted to give more evidence for pathogenesis of SVCS and provide guidance in treatment planning. All in all, the diagnosis of SVCS was established based on history, clinical presentations, and auxiliary examination, especially IVUS and the prognosis provided further evidence.

Previous studies have mentioned that SVCS was associated with a low misdiagnosis rate, and SVCS was usually diagnosed based on clinical manifestations and signs, along with various imaging techniques. However, it should be noted that the cause of SVCS can vary case-by-case, with 75% and 15% cases caused by lung cancer (small cell lung cancer based) and lymphoma, respectively. The remaining cases were caused by thymoma, germ cell tumors, breast cancer, thyroid cancer, esophageal cancer, and so on.^[11]^ Currently, some additional emerging conditions, intravascular devices like pacemaker lead implantation, hemodialysis,^[2]^ and central venous catheter placement, may contribute a lot to venous thromboses.^[12]^

Treatment strategies include steroid therapy, radiotherapy, chemotherapy, endovascular treatment, surgery, or any combination of these treatments.^[11]^ Steroids are used routinely in the management of SVCS although the effectiveness of steroids remains uncertain.^[13]^ Radiotherapy and chemotherapy are effective treatments for SVCS and should be used judiciously following the establishment of proper histopathological diagnosis and stage of the disease.^[11]^ Endovascular treatment may provide rapid symptomatic relief in patients with suspected malignant SVCS, and it has become the first-line treatment in recent years.^[14]^ Moreover, resection and reconstruction surgery of the SVCS system is feasible, while the postoperative antithrombotics, such as intravenous heparin, antiplatelet, or oral anticoagulant, are not always necessary.^[15]^ In the current case, the patient abandoned treatment and eventually died.

The induction of spinal venous obstruction due to spinal cord venous return alteration as the first symptom of SVCS is rare and to the best of our knowledge, this is the first reported case.^[16]^ To avoid misdiagnosis of spinal venous obstruction induced by SVCS, clinicians should be reminded that SVCS can induce systemic and spinal cord diseases through affecting the venous return. Clinicians should also raise awareness of this disease and pay close attention to each patient's history and comprehensively analyze the physical examination to reduce the possibility of misdiagnosis.

## 5 Summary

We reported a rare case of SVCS manifesting as spinal vein obstruction. The obstruction of the vertebral venous system returning may explain the mechanism to some extent, which is not fully understood. Further studies are needed to reveal the mechanism for SVCS inducing spinal veins obstruction and to explore whether SVCS patients with and without vascular spinal cord obstruction have different prognoses.

## References

[1] Ho YJ Yeh CH Lai CC ExPRESS miniature glaucoma shunt for intractable secondary glaucoma in superior vena cava syndrome: a case report. BMC Ophthalmol 2016;16:125. 27461379 10.1186/s12886-016-0301-6 PMC4962438

[2] Agarwal AK Khabiri H Haddad NJ Complications of vascular access: superior vena cava syndrome. Am J Kidney Dis 2017;69:309–13. 27866966 10.1053/j.ajkd.2016.08.040

[3] Straka C Ying J Kong FM Review of evolving etiologies, implications and treatment strategies for the superior vena cava syndrome. Springerplus 2016;5:229. 27026923 10.1186/s40064-016-1900-7 PMC4771672

[4] Schepers-Bok R Mallens WM Obstruction of the superior vena cava due to aortic dissection: CT findings of collateral venous flow via the bronchial veins. Eur Radiol 1996;6:753–5. 8934145 10.1007/BF00187684

[5] Burtis MT Ulmer JL Miller GA Intradural spinal vein enlargement in craniospinal hypotension. AJNR Am J Neuroradiol 2005;26:34–8. 15661695 PMC7975033

[6] Vuong SM Jeong WJ Morales H Vascular diseases of the spinal cord: infarction, hemorrhage, and venous congestive myelopathy. Semin Ultrasound CT MR 2016;37:466–81. 27616317 10.1053/j.sult.2016.05.008

[7] Gillilan LA Veins of the spinal cord. Anatomic details; suggested clinical applications. Neurology 1970;20:860–8. 4989859 10.1212/wnl.20.9.860

[8] Paksoy Y Gormus N Epidural venous plexus enlargements presenting with radiculopathy and back pain in patients with inferior vena cava obstruction or occlusion. Spine (Phila Pa 1976) 2004;29:2419–24. 15507805 10.1097/01.brs.0000144354.36449.2f

[9] Bashist B Parisi A Frager DH Abdominal CT findings when the superior vena cava, brachiocephalic vein, or subclavian vein is obstructed. AJR Am J Roentgenol 1996;167:1457–63. 8956577 10.2214/ajr.167.6.8956577

[10] Gill AE Ciszak T Braun H Intravascular ultrasound versus digital subtraction angiography: direct comparison of intraluminal diameter measurements in pediatric and adolescent imaging. Pediatr Radiol 2017;47:450–7. 28102453 10.1007/s00247-016-3771-z

[11] Talapatra K Panda S Goyle S Superior vena cava syndrome: a radiation oncologist's perspective. J Cancer Res Ther 2016;12:515–9. 27461602 10.4103/0973-1482.177503

[12] Shatila W Almanfi A Massumi M Endovascular treatment of superior vena cava syndrome via balloon-in-balloon catheter technique with a palmaz stent. Tex Heart Inst J 2016;43:520–3. 28100973 10.14503/THIJ-15-5479 PMC5179159

[13] Rowell NP Gleeson FV Steroids, radiotherapy, chemotherapy and stents for superior vena caval obstruction in carcinoma of the bronchus. Cochrane Database Syst Rev 2001;CD001316. 11687105 10.1002/14651858.CD001316

[14] Andersen PE Duvnjak S Palliative treatment of superior vena cava syndrome with nitinol stents. Int J Angiol 2014;23:255–62. 25484557 10.1055/s-0034-1383432 PMC4244246

[15] Oizumi H Suzuki K Banno T Patency of grafts after total resection and reconstruction of the superior vena cava for thoracic malignancy. Surg Today 2016;46:1421–6. 27300545 10.1007/s00595-016-1347-z

[16] Carter BW Erasmus JJ Acute thoracic findings in oncologic patients. J Thorac Imaging 2015;30:233–46. 25803363 10.1097/RTI.0000000000000148

